# Validation and implementation of array comparative genomic hybridisation as a first line test in place of postnatal karyotyping for genome imbalance

**DOI:** 10.1186/1755-8166-3-9

**Published:** 2010-04-15

**Authors:** Joo Wook Ahn, Kathy Mann, Sally Walsh, Marwa Shehab, Sarah Hoang, Zoe Docherty, Shehla Mohammed, Caroline Mackie Ogilvie

**Affiliations:** 1 Cytogenetics Department, Guy's & St Thomas' NHS Foundation Trust, London SE1 9RT, UK; 2 Cytogenetics Department, GSTS Pathology, London SE1 9RT, UK; 3 Human Cytogenetics, National Research Center, Cairo, 12311, Egypt; 4 Clinical Genetics Department, Guy's & St Thomas' NHS Foundation Trust, London SE1 9RT, UK

## Abstract

**Background:**

Several studies have demonstrated that array comparative genomic hybridisation (CGH) for genome-wide imbalance provides a substantial increase in diagnostic yield for patients traditionally referred for karyotyping by G-banded chromosome analysis. The purpose of this study was to demonstrate the feasibility of and strategies for, the use of array CGH in place of karyotyping for genome imbalance, and to report on the results of the implementation of this approach.

**Results:**

Following a validation period, an oligoarray platform was chosen. In order to minimise costs and increase efficiency, a patient/patient hybridisation strategy was used, and analysis criteria were set to optimise detection of pathogenic imbalance. A customised database application with direct links to a number of online resources was developed to allow efficient management and tracking of patient samples and facilitate interpretation of results. Following introduction into our routine diagnostic service for patients with suspected genome imbalance, array CGH as a follow-on test for patients with normal karyotypes (n = 1245) and as a first-line test (n = 1169) gave imbalance detection rates of 26% and 22% respectively (excluding common, benign variants). At least 89% of the abnormalities detected by first line testing would not have been detected by standard karyotype analysis. The average reporting time for first-line tests was 25 days from receipt of sample.

**Conclusions:**

Array CGH can be used in a diagnostic service setting in place of G-banded chromosome analysis, providing a more comprehensive and objective test for patients with suspected genome imbalance. The increase in consumable costs can be minimised by employing appropriate hybridisation strategies; the use of robotics and a customised database application to process multiple samples reduces staffing costs and streamlines analysis, interpretation and reporting of results. Array CGH provides a substantially higher diagnostic yield than G-banded chromosome analysis, thereby alleviating the burden of further clinical investigations.

## Background

Karyotype analysis of G-banded chromosomes is the cytogenetic standard for the detection of copy number imbalance across the genome, or balanced chromosome rearrangements, in children with such features as idiopathic developmental delay, learning difficulties, congenital abnormalities or autism. However, this technique has a resolution of only 3-5 Mb, and interpretation is operator-dependent, requiring highly trained and specialised staff to carry out the analysis. Higher resolution targeted tests such as fluorescence in situ hybridisation (FISH) [1] and multiplex ligation-dependent probe amplification (MLPA) [2,3] have, over the years, been added to the cytogenetic repertoire in order to increase the diagnostic yield in this group of patients.

Comparative Genomic Hybridisation (CGH) was first introduced in the early 1990s for the detection of DNA amplification in tumours [4], and used metaphase spreads as hybridisation targets. The development of array CGH, where short stretches of DNA on glass slides are used as targets instead of metaphase spreads, increased the resolution of the CGH approach and allowed the implementation of this test for more wide-spread use, particularly in tumour cytogenetics [5,6].

Array CGH has the potential to deliver a higher resolution test compared with the 3-5 Mb limit of G-banded chromosome analysis, and has added advantages of objectivity and high throughput. However, the introduction of this test into constitutional diagnostic cytogenetic services has been slow, mainly due to the expense of the consumables and to the wide-spread and established acceptance of karyotype analysis as the first-line test for genome-wide copy-number imbalance.

We have validated array CGH by testing and comparison of different platforms and hybridisation and analysis strategies, and introduced the validated protocol as a first-line test for patients with suspected copy number imbalance. Here we describe our validation studies, and the results of our diagnostic service testing from 04/2008 to 12/2009.

## Methods

### Patients

In the initial, validation, phase of this study (06/2006 to 03/2008), DNA from patients with known abnormalities, detected by G-banded chromosome analysis, was tested to confirm and validate the array procedure. In addition, patients considered likely to have submicroscopic chromosome imbalance, but with normal karyotypes, were selected and referred by our Clinical Genetics team. Results were reported as research findings only. When array CGH was introduced as a validated procedure (04/2008), testing was still restricted to those referred through our clinic following a stringent gate keeping of any requests. For these patients, formal diagnostic reports were issued. Genetic testing in the UK is funded by the National Health Service (NHS), with testing approved and commissioned by local NHS genetics commissioning bodies, based on evidence of test cost-effectiveness; following costings approval by our NHS genetics Commissioners, array CGH was introduced as a first line test in place of karyotype analysis on 05/2009, and was then applied to all samples from our region with referral indications suggesting copy number imbalance.

### Array platforms

#### BAC arrays

VIB 1 Mb (MicroArray Facility, VIB, Belgium) and Cytochip (BlueGnome, UK).

#### Oligonucleotide arrays

Agilent (USA) 4 × 44 K platform with catalogue design 014950 and Wessex NGRL design 017457 (a modified catalogue design shifting probe coverage away from cancer-related regions and towards constitutional syndromes http://www.ngrl.org.uk/wessex/arraycgh.htm.

### DNA extraction and QC

DNA was extracted from blood samples in EDTA using the Chemagic Automated DNA Separation System (Chemagen, Germany) following the manufacturer's instructions. All DNA was quantified by spectrophotometry (Nanodrop, USA) and checked for degradation on an agarose gel. Degraded DNA was not used; new samples were requested for these cases.

### Labelling DNA for array CGH

DNA (1 μg) was labelled using CGH Labelling Kit for BAC Arrays (Enzo Life Sciences, USA) or CGH Labelling Kit for Oligo Arrays (Enzo Life Sciences, USA) as appropriate, following the manufacturer's instructions.

### Purification and QC of labelled DNA

Labelled DNA was purified post- labelling using QIAquick PCR Purification Kit (Qiagen, USA) following the manufacturer's instructions. Labelling efficiency and yield was assessed by spectrophotometry (Nanodrop, USA).

### Processing arrays

Hybridisation, washing and scanning of arrays was performed following the manufacturers' protocols.

### Hybridisation strategies

Three different strategies were considered:

i) "dye swap" - hybridisation of patient DNA against control DNA, with a repeat assay but with labelling in opposite colours. Two arrays were required for each patient sample, and this strategy gave confidence that the array findings were not artefactual, and controlled for bias in cyanine (Cy) dye labelling reaction efficiencies.

ii) "loop" [7]- three patient samples labelled in each of two colours, then each sample hybridised against the other two patient samples. Only one array was required for each patient sample, and if the two labelling runs were carried out on separately-accessed DNA aliquots, then no confirmation of sample identity was required. This strategy also gave confidence that the array findings were not artefactual, and controlled for bias in Cy dye labelling reaction efficiencies.

iii) "patient/patient" - differentially-labelled patients hybridised against each other. Patients were phenotype mismatched as any shared imbalance would not be detected. Cy dye ratios informed "ownership" of any detected imbalance (log2 ratios of -1 and 0.6 indicate deletion and duplication respectively), which was confirmed by a second array or by MLPA for any syndromic imbalance covered by MLPA kits P064 and P245 (~6% of cases; MRC-Holland, Netherlands). This second test also confirmed sample identity. Therefore the first round of array testing required half an array; for each detected imbalance (in approximately 25% of patients), a further half array is required. This therefore resulted in an overall expenditure of 5/8 of an array per patient, providing substantial cost savings over other approaches.

### Array data analysis

All arrays were scanned at 5 μm resolution (Agilent, USA).

#### BAC arrays

Image quantification, array quality control and aberration detection were performed using BlueFuse software (BlueGnome, UK). 95% of array data was required to pass QC. Samples failing this QC were repeated. A normal range for log2 fluorescence ratios was set to 0 +/- 4 SD.

#### Oligo arrays

Image quantification, array quality control and aberration detection were performed using Feature Extraction and DNA Analytics software packages (Agilent, USA) for oligo arrays. Manufacturer's recommendations were followed. 95% of array data was required to pass QC. Samples failing this QC were repeated. Two rounds of aberration calling were employed, ADM-2 algorithm at threshold 6 (with a 3 probe sliding window providing a mean detection interval of 200 kb) for detection of copy number imbalance, and ADM-1 algorithm at threshold 6 for detection of low-level mosaicism. Each called aberration was assessed by an analyst to provide a further measure of quality, to examine the genomic context of the aberration and to identify any specific genes/regions where further investigations might be required.

### Fluorescence in situ hybridisation

BAC array findings were confirmed by fluorescence in situ hybridisation (FISH) using the same clones (Bluegnome, UK and BACPAC, Sanger Institute, UK) as on the array [8].

### Multiplex ligation-dependent probe amplification

MLPA was used to confirm imbalance of syndromic regions represented in MLPA kits P064 and P245 (MRC-Holland, Netherlands). Furthermore, inheritance studies were performed by incorporating custom-designed MLPA probes, specific for loci within the region of imbalance, into MLPA kit P200 (MRC-Holland, Netherlands). Custom MLPA probes were designed using the H-MAPD tool [9]. All MLPA reactions were performed following the manufacturer's instructions.

### QF-PCR

Quantitative Fluorescence PCR was carried out as previously described [10]

### Reporting

Imbalances of regions represented in the Database of Genomic Variants (DGV) [11] as being present in the normal population (in at least three non-BAC based studies) were recorded but not reported. All other imbalances outside areas of known syndromic imbalance were reported as preliminary findings, with a request for parental blood samples to establish inheritance. Inherited imbalances were reported as probably benign, although "unusual features shared by the proband and carrier parent may be associated with the imbalance". Genes in regions of *de novo *imbalance were described if considered relevant to the referral indication. The average reporting time for first-line tests was 25 days from receipt of sample.

### Final diagnostic service protocol

**Figure 1:**
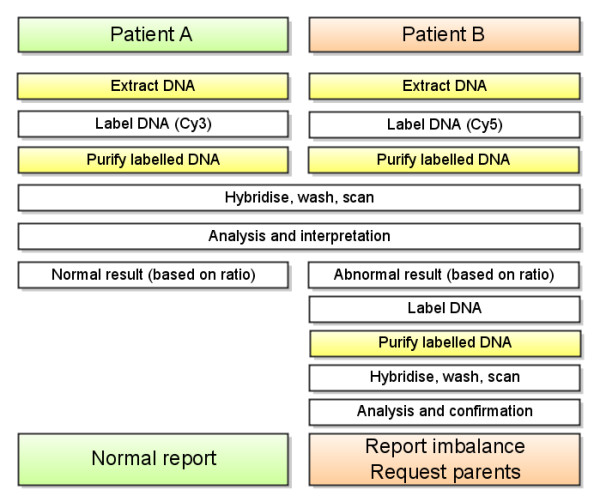
**Array CGH diagnostic service workflow**. This example illustrates the diagnostic workflow for two patients, one of whom has a previously undetected imbalance of potential clinical significance. Yellow shading signifies automated procedures (we plan to also automate labelling, hybridisation and washing).

Patient samples were processed as detailed above, on the Agilent 44 K platform (Wessex NGRL design 017457). A patient/patient hybridisation strategy provided substantial consumable and labour savings while maintaining diagnostic yield. Figure 1 shows an example workflow for two patients, one of whom carries an imbalance, that are hybridised against each other. For findings not present in DGV, Cy3/Cy5 signal ratios were used to determine which hyb partner carried the imbalance. A second follow-up array was used to confirm imbalance in this patient, before reporting both hyb partners. Once parental samples were received, custom MLPA or array testing were used as appropriate for inheritance studies.

## Results

### Karyotype audit

Array CGH testing does not detect balanced rearrangements; we therefore audited a 10-year period of karyotype results from our laboratory, which showed that of a total of 36,663 postnatal blood samples received, 4 (0.0001%) were found to have de novo reciprocal translocations.

### Array validation

Both BAC array and oligoarray validation included "self-self" hybridisations and DNA from cases of known imbalance including trisomies for chromosomes 13 and 18, and deletions and duplications ranging from 0.5-7.1 Mb. Both array platforms gave the expected results from these validation experiments. Further to these known abnormalities, a number of patients with normal karyotypes but considered likely to carry imbalance were also tested. Where imbalance was detected by BAC array, FISH with the abnormal clones was carried out where possible. For 3/16 (18%) single clone results, the FISH result was not concordant with the BAC array findings. This was considered to be due to the differing sensitivities of the techniques. All larger imbalances detected by BAC arrays were confirmed by FISH. Abnormalities detected on oligoarrays were followed up by a variety of techniques (MLPA, QF-PCR, retrospective G-banded chromosome analysis) in 68 cases; no discrepancies were found.

42 patients (with normal karyotypes) were tested on both BAC and oligo array platforms. 30 of these gave normal results on both platforms while 6 gave abnormal results on both platforms. In these concordant cases (Table 1), the resolution of the breakpoints given by the oligo arrays was higher than that by the BAC arrays, due to higher probe density across the genome and the smaller size of the oligo probes. The remaining 6 discordant results (Table 2) all demonstrate imbalances that were not detected by the BAC platform. Most notable is a patient with imbalance of the 16p11.2 autism susceptibility locus [12].

**Table 1:** Patients tested on BAC and oligo array platforms with concordant results.

Patient	BAC array result	Oligo array result
1	2q33.1 × 3 (0.3-1.7 Mb)	2q33.1 × 3 (0.6 Mb)

2	7p12.1 × 3 (0.2-2.6 Mb)	7p12.1 × 3 (0.8 Mb)

3	14q12 × 3 (0.2-2.1 Mb)	14q12 × 3 (0.4 Mb)

4	19q12 × 1 (0.2-1.8 Mb)	19q12 × 1 (0.7 Mb)

5	Xp22.12p22.13 × 3 (0.8-2.0 Mb)	Xp22.12p22.13 × 3 (0.8 Mb)

6	Xp11.22 × 2 (0.2-1.6 Mb)	Xp11.22 × 2 (0.3 Mb)

**Table 2:** Patients tested on BAC and oligo array platforms with discordant results.

Patient	BAC array result	Oligo array result
1	None	1q44 × 3 (0.3 Mb)

2	None	9q33 × 1 (0.2 Mb)

3	19q13 × 3 (0.2 Mb)	14q32 × 3 (0.2 Mb), 19q13 × 3 (0.1 Mb)

4	None	16p11 × 1 (3.0 Mb)

5	None	16p11 × 3 (0.2 Mb)

6	None	17p13 × 3 (0.5 Mb)

### Diagnostic service

A total of 2,414 patients were tested as part of the diagnostic service. Overall, 24% of patients tested had copy number variants (CNVs) not represented in the DGV, and therefore considered to be potentially pathogenic (see Tables 3 and 4). These imbalances comprised 374 deletions (range 2 kb to 19.215 Mb), 300 duplications (range 12 kb to 34.086 Mb) and 27 triplications (range 26 kb to 1.786 Mb). The smallest imbalance was a 2 kb (3 probes) intragenic deletion within ZNF519, a zinc finger gene involved in regulation of transcription. Parental samples for inheritance studies of this imbalance have not yet been received.

**Table 3:** Results from diagnostic service.

	All oligo arrays (service)	Oligo arrays following normal karyotype	Oligo arrays as first line test
Total patients	2414	1245	1169

Abnormal patients	585 (24%)	325 (26%)	260 (22%)

- inherited	169	97	72

- de novo	63	52	11

- unknown inheritance	353	176	177

**Table 4:** Imbalances detected during diagnostic service (some patients carried more than one region of imbalance).

	All oligo arrays (service)	Oligo arrays following normal karyotype	Oligo arrays as first line test
Total imbalances	715	404	311

- deletion	374	223	151

- duplication	300	155	145

- triplication	27	18	9

- marker	1		1

- whole chromosome	13 (incl. 7 mosaic)	8 (incl. 3 mosaic)	5 (incl. 4 mosaic)

**Figure 2:**
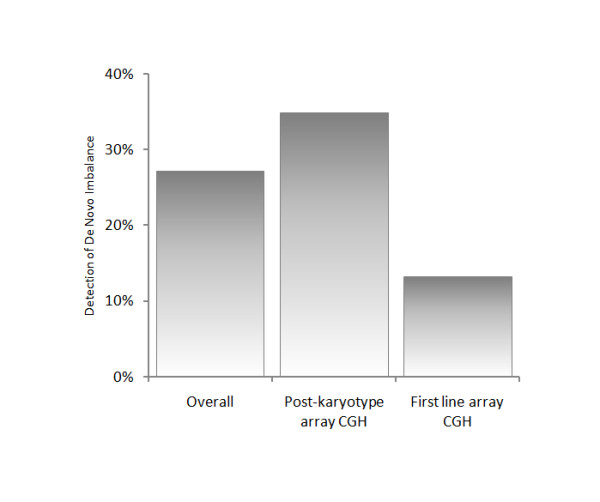
**Comparison of *de novo *imbalance detection rates**. Proportion of *de novo *findings in first line array CGH and post-normal karyotype array CGH cohorts. Higher rates of *de novo *findings in the post-normal karyotype cohort reflect the stringent clinical selection of these patients; these patients would have been diagnosed at an earlier stage had array CGH been carried out as the first line test.

All patients with imbalance not represented in the DGV were followed up by other techniques or repeat arrays prior to reporting. Parental samples were requested in all cases, but only received for 40%. These inheritance studies showed that *de novo *imbalances accounted for 35% of findings for patients tested after a normal karyotype, and 13% of findings from first line tests (Figure 2). At least 89% of the abnormalities detected by first line testing would not have been detected by karyotype analysis (assuming a 3 Mb detection threshold for imbalance detection by G-banded chromosome analysis). Overall, 14% of imbalances detected fell within known sites of recurrent microdeletion/duplication.

## Discussion

Array CGH testing does not detect balanced rearrangements, and therefore may miss a clinically significant karyotype where a de novo balanced rearrangement may be disrupting gene function. We therefore carried out an audit of postnatal karyotype results at our Centre which showed that over this period 0.0001% of samples had a de novo reciprocal translocation. Inherited translocations would be considered incidental findings, which, although potentially of value for reproductive counselling for the parents, would not be relevant to the proband's referral. The increase in detection rate of chromosome imbalance using our strategy is therefore a major benefit, whereas the number of clinically significant chromosome rearrangements that remain undetected is extremely low.

The validation study indicated that the oligonucleotide approach was robust and cost-effective, as well as having a higher resolution than BAC arrays (see Tables 1 and 2), with no false positive findings when followed up with different methodologies, or false negative findings when samples with abnormal karyotypes were tested. The choice of the 4 × 44 K array platform was pragmatic, with the aim of maximising detection of clinically significant imbalances whilst minimising costs.

This approach was therefore introduced as a diagnostic test in May 2008, in tandem with a comprehensive education initiative for local users of the service. Since then, 2,414 patients have been tested, with an overall imbalance detection rate of 24% (excluding previously published population polymorphisms (see above)). Previously published reports of detection rates in cohorts of patients with congenital anomalies, mental disability or dysmorphism, are heterogeneous in design and application; those reporting cohorts of ≥ 100 patients include those using tiling-path BAC arrays [13], 1 Mb BAC arrays [14-16], targeted BAC arrays [17-19], and oligonucleotide arrays [20-22]. The majority of patients in these cohorts had normal karyotypes, and some had also had subtelomeric imbalance excluded using either FISH or MLPA. The overall imbalance detection rates ranged from ~8% (using targeted BAC arrays) [17] to 20% (using 1 Mb BAC arrays [14] or oligonucleotide arrays [22]). These previous studies therefore indicate that the protocols described here, including the patient/patient hybridisation strategy, did not compromise the diagnostic yield in this cohort.

Figure 2 shows that *de novo *abnormalities were less prevalent when testing was carried out as a first line test. This is likely to be due to stringent selection for post-karyotype array CGH of patients likely to carry submicroscopic imbalance, carried out by expert dysmorphologists and clinical geneticists. The cohort of patients tested by array CGH as a first line test included those with referrals more likely to be associated with polygenic and multifactorial conditions such as autism and Attention Deficit Hyperactivity disorder, where genome imbalance is less likely to be found, but needs to be excluded. *De novo *abnormalities are generally considered likely to be pathogenic and causative of the patient's phenotype, whilst it has been suggested that inherited imbalances should be classified as "false positive" findings [23]. However, in some families, the carrier parent may show mild manifestation of the clinical phenotype affecting the child, and expert phenotypic assessment of the carrier parent is necessary to ascertain this; indeed, in our patient group we have found families where the imbalance was present in more than one other family member, all of whom had phenotypes that were very much milder than that of the proband. The possibility that a deletion unmasks a recessive allele in the proband is another reason for careful consideration of the gene content of inherited as well as *de novo *imbalances. As more information on the function and copy-number sensitivity of genes is accumulated, the interpretation of such findings is likely to become more straightforward.

### Rationale for service strategy

The protocols described here were developed with the aim of providing an array CGH service as a first line test with little or no additional cost over and above that of our current karyotype analysis. This cost includes confirmatory testing of detected imbalance and subsequent inheritance studies.

The following factors contributed to reducing costs and increasing efficiency of array CGH testing:

i) Patient/patient hybridisation: this strategy halved consumable costs, and increased the efficiency of analysis (for normal arrays, only one analysis was required for two patients). The disadvantage of this approach is that, were patients with the same imbalance to be hybridised against each other, the abnormality would not be detected (two patients with deletion and duplication of the same region respectively would be detected). Our protocol therefore included a careful consideration of patient referral information when constructing run sheets for hybridisation (phenotype mismatching), with the aim of reducing the incidence of missed abnormalities. However, with the sparse clinical information provided on many referral forms, some imbalances may have been missed. Data from our cohort suggests a less than 1 in 25,000 chance of failing to detect 16p11.2 deletion syndrome (our most common finding, present in 15 (~0.62% of patients) even without phenotype mismatching. Furthermore, the abnormality pick-up rate for array CGH testing is considerably higher than for karyotype analysis, and any abnormalities missed as a consequence of the hybridisation strategy would likely be beyond the resolution of G-banded chromosome analysis.

ii) Batch testing: processing of multiple samples for labelling, clean-up and hybridisation considerably reduced the technical time required for testing, allowing a throughput of 96 samples/week.

iii) Robotics: automating DNA purification reduced technical input time, and increased consistency of processing. We aim also to automate DNA labelling in the near future to reduce costs and reporting times further, to allow increased throughput, and to improve quality and consistency.

iv) Dedicated IT resources: a database application was designed and built during the validation phase of this study. This allowed rapid processing of information, audit, sample tracking and construction of run sheets, with click-through links to internet resources, in particular the DGV, to expedite diagnosis and interpretation of findings.

The combination of the above resources and strategies has enabled us to offer this test, including confirmatory and inheritance follow-up testing, at the same cost as karyotype analysis. We recognise that other centres will have different financial and staffing constraints and may not have access to some of the equipment and expertise required, but nevertheless we feel that aspects of this approach may be of interest to those wishing to introduce array CGH testing into their diagnostic service.

The clinical utility and cost-effectiveness of array CGH have been discussed in previous papers, which concluded that, even without the increases in efficiency described in this paper, array CGH results in a cost per diagnosis less than that for karyotype analysis [24], and predicts that array CGH is likely to become the genetic test of choice for patients with suspected copy number imbalance [25].

## Conclusions

This study demonstrates the feasibility of using oligonucleotide array CGH as a first line diagnostic test in place of G-banded chromosome analysis for genome-wide constitutional imbalance. This approach can be implemented with minimal cost implications using the protocols described. Array CGH provides a substantially higher diagnostic yield than G-banded chromosome analysis, thereby alleviating the burden of further clinical investigations, and is providing valuable information on the link between gene copy number and expression of abnormal phenotype.

## Competing interests

The authors declare that they have no competing interests.

## Authors' contributions

JWA organised the validation and diagnostic service work, built the database application, and extracted and analysed the data for the paper; KM co-wrote the grant proposal for the validation study, and participated in the validation, service development and custom MLPA follow-up strategy; SW participated in the validation and diagnostic service and design of the custom MLPA probes; MS carried out validation work on the BAC arrays; SH carried out the validation work and experiments on mosaicism detection; ZD provided advice on reporting and facilitated service implementation; SM provided clinical information and advice and facilitated service implementation; CO co-wrote the grant proposal for the validation study, devised and coordinated service strategies and wrote the paper. All authors read and approved the final manuscript.

## References

[B1] Trask BJ Fluorescence in situ hybridization: applications in cytogenetics and gene mapping Trends Genet 1991 7 149 154 2068787 10.1016/0168-9525(91)90378-4

[B2] Schouten JP McElgunn CJ Waaijer R Zwijnenburg D Diepvens F Pals G Relative quantification of 40 nucleic acid sequences by multiplex ligation-dependent probe amplification Nucleic Acids Res 2002 30 e57 10.1093/nar/gnf056 12060695 PMC117299

[B3] Ahn JW Ogilvie CM Welch A Thomas H Madula R Hills A Donaghue C Mann K Detection of subtelomere imbalance using MLPA: validation, development of an analysis protocol, and application in a diagnostic centre BMC Med Genet 2007 8 9 10.1186/1471-2350-8-9 17338807 PMC1831468

[B4] Kallioniemi OP Kallioniemi A Sudar D Rutovitz D Gray JW Waldman F Pinkel D Comparative genomic hybridization: a rapid new method for detecting and mapping DNA amplification in tumors Semin Cancer Biol 1993 4 41 46 8448377

[B5] Solinas-Toldo S Lampel S Stilgenbauer S Nickolenko J Benner A Dohner H Cremer T Lichter P Matrix-based comparative genomic hybridization: biochips to screen for genomic imbalances Genes Chromosomes Cancer 1997 20 399 407 10.1002/(SICI)1098-2264(199712)20:4<399::AID-GCC12>3.0.CO;2-I 9408757

[B6] Pinkel D Segraves R Sudar D Clark S Poole I Kowbel D Collins C Kuo WL Chen C Zhai Y High resolution analysis of DNA copy number variation using comparative genomic hybridization to microarrays Nat Genet 1998 20 207 211 10.1038/2524 9771718

[B7] Allemeersch J Van Vooren S Hannes F De Moor B Vermeesch JR Moreau Y An experimental loop design for the detection of constitutional chromosomal aberrations by array CGH BMC Bioinformatics 2009 10 380 10.1186/1471-2105-10-380 19925645 PMC2791104

[B8] Davies AF Kirby TL Docherty Z Ogilvie CM Characterization of terminal chromosome anomalies using multisubtelomere FISH Am J Med Genet A 2003 120A 483 489 10.1002/ajmg.a.20056 12884426

[B9] Zhi J Hatchwell E Human MLPA Probe Design (H-MAPD): a probe design tool for both electrophoresis-based and bead-coupled human multiplex ligation-dependent probe amplification assays BMC Genomics 2008 9 407 10.1186/1471-2164-9-407 18783624 PMC2547856

[B10] Ogilvie CM Donaghue C Fox SP Docherty Z Mann K Rapid prenatal diagnosis of aneuploidy using quantitative fluorescence-PCR (QF-PCR) J Histochem Cytochem 2005 53 285 288 10.1369/jhc.4B6409.2005 15750003

[B11] Iafrate AJ Feuk L Rivera MN Listewnik ML Donahoe PK Qi Y Scherer SW Lee C Detection of large-scale variation in the human genome Nat Genet 2004 36 949 951 10.1038/ng1416 15286789

[B12] Fernandez BA Roberts W Chung B Weksberg R Meyn S Szatmari P Joseph-George AM Mackay S Whitten K Noble B Phenotypic Spectrum Associated with De Novo and Inherited Deletions and Duplications at 16p11.2 in Individuals Ascertained for Diagnosis of Autism Spectrum Disorder J Med Genet 2009 19755429 10.1136/jmg.2009.069369

[B13] de Vries BB Pfundt R Leisink M Koolen DA Vissers LE Janssen IM Reijmersdal S Nillesen WM Huys EH Leeuw N Diagnostic genome profiling in mental retardation Am J Hum Genet 2005 77 606 616 10.1086/491719 16175506 PMC1275609

[B14] Menten B Maas N Thienpont B Buysse K Vandesompele J Melotte C de Ravel T Van Vooren S Balikova I Backx L Emerging patterns of cryptic chromosomal imbalance in patients with idiopathic mental retardation and multiple congenital anomalies: a new series of 140 patients and review of published reports J Med Genet 2006 43 625 633 10.1136/jmg.2005.039453 16490798 PMC2564583

[B15] Baris HN Tan WH Kimonis VE Irons MB Diagnostic utility of array-based comparative genomic hybridization in a clinical setting Am J Med Genet A 2007 143A 2523 2533 10.1002/ajmg.a.31988 17910064

[B16] Pickering DL Eudy JD Olney AH Dave BJ Golden D Stevens J Sanger WG Array-based comparative genomic hybridization analysis of 1176 consecutive clinical genetics investigations Genet Med 2008 10 262 266 10.1097/GIM.0b013e31816b64ad 18414209

[B17] Sharp AJ Hansen S Selzer RR Cheng Z Regan R Hurst JA Stewart H Price SM Blair E Hennekam RC Discovery of previously unidentified genomic disorders from the duplication architecture of the human genome Nat Genet 2006 38 1038 1042 10.1038/ng1862 16906162

[B18] Lu X Shaw CA Patel A Li J Cooper ML Wells WR Sullivan CM Sahoo T Yatsenko SA Bacino CA Clinical implementation of chromosomal microarray analysis: summary of 2513 postnatal cases PLoS One 2007 2 e327 10.1371/journal.pone.0000327 17389918 PMC1828620

[B19] Shaffer LG Bejjani BA Torchia B Kirkpatrick S Coppinger J Ballif BC The identification of microdeletion syndromes and other chromosome abnormalities: cytogenetic methods of the past, new technologies for the future Am J Med Genet C Semin Med Genet 2007 145C 335 345 10.1002/ajmg.c.30152 17910076

[B20] Fan YS Jayakar P Zhu H Barbouth D Sacharow S Morales A Carver V Benke P Mundy P Elsas LJ Detection of pathogenic gene copy number variations in patients with mental retardation by genomewide oligonucleotide array comparative genomic hybridization Hum Mutat 2007 28 1124 1132 10.1002/humu.20581 17621639

[B21] Shen Y Irons M Miller DT Cheung SW Lip V Sheng X Tomaszewicz K Shao H Fang H Tang HS Development of a focused oligonucleotide-array comparative genomic hybridization chip for clinical diagnosis of genomic imbalance Clin Chem 2007 53 2051 2059 10.1373/clinchem.2007.090290 17901113

[B22] Jaillard S Drunat S Bendavid C Aboura A Etcheverry A Journel H Delahaye A Pasquier L Bonneau D Toutain A Identification of gene copy number variations in patients with mental retardation using array-CGH: Novel syndromes in a large French series Eur J Med Genet 2009 10.1016/j.ejmg.2009.10.002 19878743

[B23] Sagoo GS Butterworth AS Sanderson S Shaw-Smith C Higgins JP Burton H Array CGH in patients with learning disability (mental retardation) and congenital anomalies: updated systematic review and meta-analysis of 19 studies and 13,926 subjects Genet Med 2009 11 139 146 10.1097/GIM.0b013e318194ee8f 19367186

[B24] Wordsworth S Buchanan J Regan R Davison V Smith K Dyer S Campbell C Blair E Maher E Taylor J Knight SJ Diagnosing idiopathic learning disability: a cost-effectiveness analysis of microarray technology in the National Health Service of the United Kingdom Genomic Med 2007 1 35 45 10.1007/s11568-007-9005-6 18923927 PMC2276893

[B25] Edelmann L Hirschhorn K Clinical utility of array CGH for the detection of chromosomal imbalances associated with mental retardation and multiple congenital anomalies Ann N Y Acad Sci 2009 1151 157 166 10.1111/j.1749-6632.2008.03610.x 19154522

